# Mild and Efficient Winterfeldt Oxidation of 1,2,3,4-Tetrahydro-γ-carbolines for the Synthesis of Dihydropyrrolo[3,2-*b*]-quinolones and Pyrrolo[3,2-*b*]quinolones

**DOI:** 10.3390/molecules17021177

**Published:** 2012-01-30

**Authors:** Rong Sheng, Jiangwei Zhu, Yongzhou Hu

**Affiliations:** ZJU-ENS Joint Laboratory of Medicinal Chemistry, Zhejiang University, Hangzhou 310058, China

**Keywords:** Winterfeldt oxidation, 1,2,3,4-tetrahydro-γ-carbolines, dihydropyrrolo[3,2-*b*]-quinolones, pyrrolo[3,2-*b*]quinolones

## Abstract

The Winterfeldt oxidation (NaOH, DMF, air, rt) of substituted 1,2,3,4-tetrahydro-γ-carbolines has been developed, which provides a convenient and efficient method for the synthesis of the corresponding dihydropyrrolo[3,2-b]quinolones in moderate to excellent yields (38–94%). The generality and substrate scope of this reaction are explored and a possible mechanism is proposed. The results imply that electron-withdrawing groups on N^2^ of tetrahydro-γ-carbolines and N^5^-H are necessary. The synthesis of 5 or 7-substituted pyrrolo[3,2-*b*]quinolones in near quantitative yields was also achieved through deprotection and aromatization of N^1^-*b*oc-dihydropyrrolo[3,2-*b*]quinolones.

## 1. Introduction

Fused tricyclic and tetracyclic quinolone scaffolds have been reported to possess many biological properties. For example, pyrroloquinolone **1** is a highly potent and selective PDE5 inhibitor [1], 2,3-dihydro-1H-cyclopenta[*b*]quinolin-9(4H)-one derivative **2** shows antimalarial activity [2], and cryptolepine analogue **3** displays antiplasmodial activity [3]. Among all the reported synthetic methods for the construction of fused quinolone scaffolds, the biomimetic Winterfeldt oxidation has attracted much interest because of its simple procedure and widely available substrates [4]. Particularly, the Winterfeldt oxidation of 1,2,3,4-tetrahydro-β-carbolines with different reagent systems including NaH/DMF/O_2_ [5], *t*-BuOK/DMF/O_2_ [6], and KO_2_/18-crown-6/DMF [7,8] was extensively studied in the past few years, which has not only provided an efficient method for the preparation of pyrroloquinolone derivatives such as **1** and **4**, but also for crucial indole-quinolone transformations in the total synthesis of potent antitumor agent (±)-camptothecin **5** [9] and TNF production inhibitor (S)-(−)-quinolactacin B **6** [10,11].

**Figure 1:**
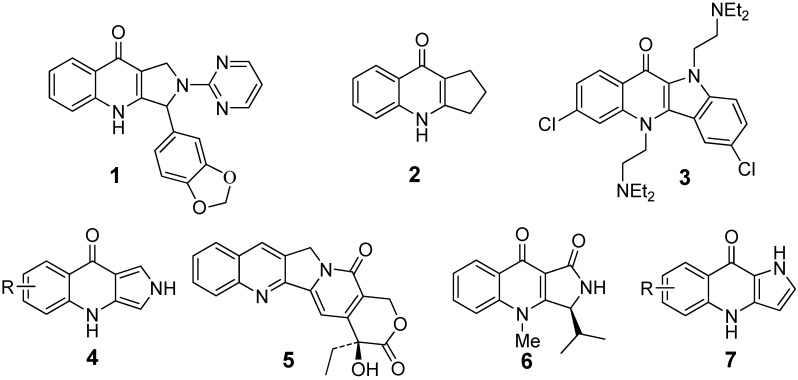
Structures of compounds with fused quinolone scaffolds.

As bioisosteres of pyrrolo[3,4-*b*]quinolones **4**, pyrrolo[3,2-*b*]quinolinones **7** (Figure 1) were first synthesized by Mentel via solid phase Witkop-Winterfeldt oxidation in 2009 [12]. Polymer-bound 2-tosyl-1,2,3,4-tetrahydro-γ-carbolines were converted to ketolactams with ozone in CH_2_Cl_2_ at −78 °C, followed by refluxing in Et_3_N/DMF overnight to yield the target compounds. More recently, the same group reported the scalable solution phase synthesis of pyrrolo[3,4-*b*]quinolones in low to moderate yield (36–60%) with the same two-step method [13]. However, only the 2-tosyl-1,2,3,4-tetrahydro-γ-carbolines were used as the substrates, so the generality and substrate scope of the reaction have not been extensively explored, and the harsh reaction conditions, tedious procedure with a low to moderate yield of product may limit its wider application. In continuation of our ongoing studies on the synthesis and reactions of γ-carboline derivatives, we report herein the Winterfeldt oxidation of substituted 1,2,3,4-tetrahydro-γ-carbolines under mild reaction conditions (NaOH, DMF, air, rt) [14,15]. The optimized methodology provided for the rapid construction of a variety of substituted dihydropyrrolo[3,2-*b*]quinolones and pyrrolo[3,2-*b*] quinolones in good to excellent yields. 

## 2. Results and Discussion

**Scheme 1:**
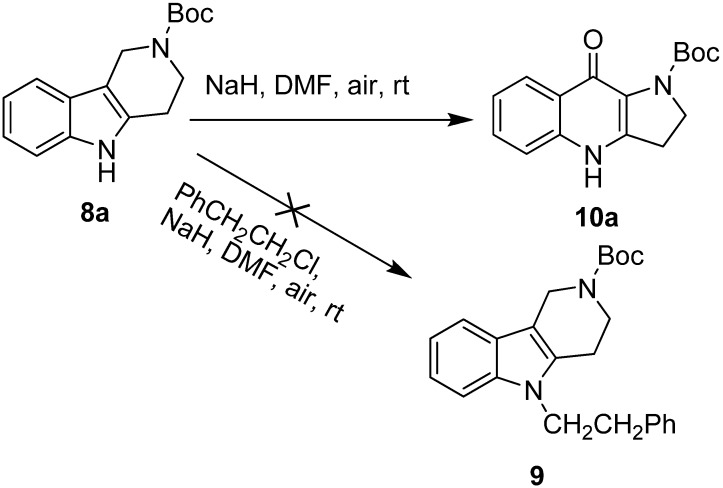
Winterfeldt oxidation of 2-Boc-1,2,3,4-tetrahydro-γ-carboline.

In our attempt to synthesize compound **9** through alkylation of 2-Boc-1,2,3,4-tetrahydro-γ-carboline **8a** with PhCH_2_CH_2_Cl in DMF using NaH as the base (Scheme 1), an unexpected product was produced in excellent yield (92%), and the structure was established as 2-Boc-dihydropyrrolo[3,2-*b*]-quinolone **10a** by analysis of the corresponding ^1^H-NMR, ^13^C-NMR and MS spectra. We found that no reaction occurred when **8a** was stirred with NaH/DMF under nitrogen. This result clearly indicated that a Winterfeldt oxidation of 2-Boc-tetrahydro-γ-1,2,3,4-tetrahydro-carboline had occurred.

Encouraged by this initial finding, we turned our attention towards the development of a convenient and efficient method on the synthesis of dihydropyrrolo[3,2-b]quinolones and pyrrolo[3,2-b]-quinolones via Winterfeldt oxidation.

As shown in Table 1, by using 2-Boc-1,2,3,4-tetrahydro-γ-carboline (**8a**) as a model compound, a survey of reaction conditions was carried out. We first examined the effect of different bases on the yields of product. The results demonstrated that *t*-BuOK, NaOCH_3_, and NaOH were all capable of promoting the Winterfeldt oxidation with almost the same yields of product as NaH, while the reaction using K_2_CO_3_ as base failed, probably due to its weak basicity (Table 1, entries 3–6).

**Table 1:** Optimization of the Winterfeldt oxidation of 2-Boc-1,2,3,4-tetrahydro-γ-carboline.

Entry	Base	Equiv of base	Solvent		Time/h	Yield (%)
1	NaH	3.0	DMF	air	4	94
2	NaOH	3.0	DMF	N_2_	2	0
3	t-BuOK	3.0	DMF	air	4	95
4	MeONa	3.0	DMF	air	4	93
5	NaOH	3.0	DMF	air	5	94
6	K_2_CO_3_	3.0	DMF	air	24	0
7	NaOH	2.0	DMF	air	5	94
8	NaOH	1.5	DMF	air	5	87
9	NaOH	2.0	DMSO	air	5	92
10	NaOH	2.0	THF	air	20	35 ^a^
11	NaOH	2.0	MeOH	air	5	0
12	NaOH	2.0	DMF	O_2_	2	94

^a^ Reflux, 42% substrate was recovered.

**Scheme 2:**
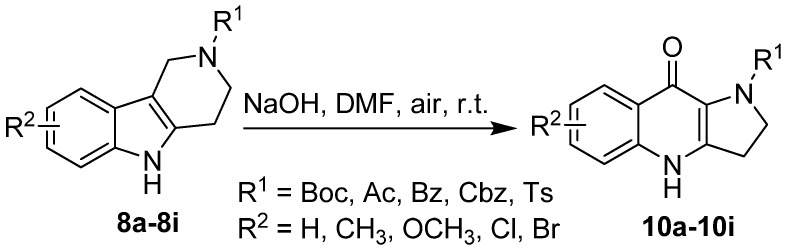
Winterfeldt oxidation of 2-substituted-1,2,3,4-tetrahydro-γ-carboline.

Because of its simple handling and wide availability, NaOH was chosen as the suitable base for the following optimization. Subsequently, the molar ratio of NaOH to substrate **8a** was examined, the results suggested that 2.0 equiv. of base was sufficient for this reaction (Table 1, entries 7, 8). A screen of solvents revealed that DMF provided the best yield, MeOH completely inhibited the oxidation, and the yield in THF was poor because of the incomplete conversion of substrate, even under reflux conditions (Table 1, entries 9–11). Usually the Winterfeldt oxidation is carried out with a base in DMF in the presence of oxygen [4,5,6], therefore, a similar reaction with a balloon containing oxygen was performed, and the reaction was complete in 2 h with the same yield as seen in the air. These results suggest that the air performs the same role as the oxygen. (Table 1, entry 12). Considering the convenience of operation and wide availability of reagents, the Winterfeldt oxidation of 2-Boc-1,2,3,4-tetrahydro-γ-carbolines was thus best run in DMF with 2.0 equiv. of NaOH at room temperature for 5 h in the presence of air (Table 1, entry 5). With the optimized reaction conditions, we next examined the generality and substrate scope of this Winterfeldt oxidation reaction. A series of 6- or 8-substituted 2-Boc-1,2,3,4-tetrahydro-γ-carbolines were employed and the diversity of substituents in the 2-position of 1,2,3,4-tetrahydro-γ-carbolines was investigated (Scheme 2).

As shown in Table 2, all the substituted 2-Boc–1,2,3,4-tetrahydro-γ-carbolines examined participate in the Winterfeldt oxidation in excellent yields, which implied that the electronic properties of different substituents in the 6- or 8-position does not affect this transformation very much (Table 2, entries 1–5).

**Table 2:** Winterfeldt oxidation of substituted 1,2,3,4-tetrahydro-γ-carbolines.

Entry	7	R^1^	R^2^	Time/h	Product/yield (%)
1	**8a**	Boc	H	5	**10a** (94)
2	**8b**	Boc	8-CH_3_	5	**10b** (92)
3	**8c**	Boc	8-OCH_3_	6	**10c** (89)
4	**8d**	Boc	8-Br	5	**10d** (92)
5	**8e**	Boc	6-Cl	6	**10e** (93)
6	**8f**	Ac	H	6	**10f** (87)
7	**8g**	Bz	H	7	**10g** (89)
8	**8h**	Cbz	H	6	**10h** (86)
9	**8i**	Ts	H	8	**10i** (38)
10	**8j**	H	H	24	－ ^a^
11	**8k**	Me	H	24	－ ^a^
12	**8l**	Bn	H	24	－ ^a^

a No desired product was obtained even with NaH/DMF/O2 or *t*-BuOK/DMF/O2.

The change of substituents in the 2-position has an obvious effect on the yields of products, since while like the 2-Boc substituted derivatives, the 2-acetyl, 2-benzoyl and 2-Cbz substituted 1,2,3,4-tetrahydro-γ-carbolines were converted into the corresponding dihydropyrrolo[3,2-b]quinolones in good yields (86–89%), the yield of the 2-tosyl-dihydropyrrolo[3,2-b]quinolones was only 38%, close to the literature value, perhaps due to the formation of other by-products (Table 2, entries 6–9) [13]. The 2-unsubstituted, 2-methyl and 2-benzyl 1,2,3,4-tetrahydro-γ-carbolines were also evaluated in this reaction, but none of desired products were obtained, even with NaH/DMF/O_2_ and *t*-BuOK/DMF/O_2_ (Table 2, entries 10–12).

**Scheme 3:**
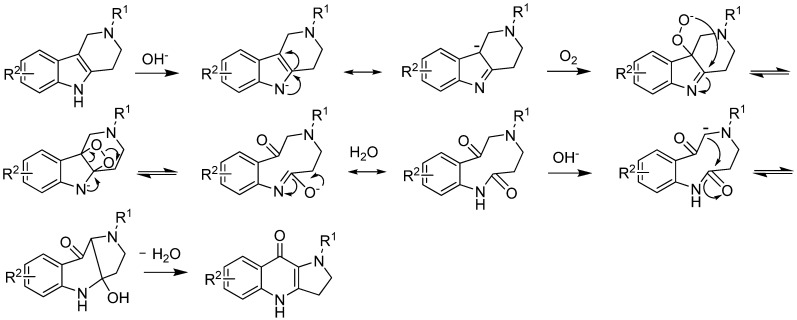
Proposed mechanism for the formation of substituted dihydropyrrolo[3,2-b]-quinolones.

All these results imply that electron-withdrawing groups on the 2-position of 1,2,3,4-tetrahydro-γ-carbolines are necessary for this variant of the Winterfeldt oxidation, which may favor the formation of ketolactam intermediates. It also shows that 2-Boc-5-methyl-1,2,3,4-tetrahydro-γ-carboline could not be converted to corresponding quinolone under the optimized conditions, which clearly indicates that prior formation of the N-anion is necessary in this reaction. On the basis of these results and previous studies by Mentel and others [4,13], a plausible mechanism for this improved Winterfeldt oxidation is proposed in Scheme 3.

**Scheme 4:**
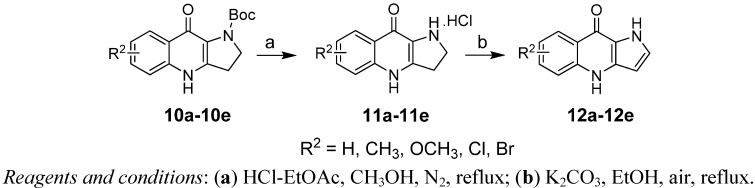
Synthesis of dihydropyrrolo[3,2-*b*]quinolones hydrochloride **11a–e** and pyrrolo[3,2-*b*]quinolones **12a–e**.

After a careful survey of reaction conditions, the substituted 2-Boc-dihydropyrrolo[3,2-*b*]-quinolones **10a–e** were refluxed in HCl-EtOAc/CH_3_OH under nitrogen to afford dihydropyrrolo-[3,2-*b*]quinolones **11a–e** in excellent yields as the corresponding hydrochloride salts. We found that the free amines of **11a–e** were unstable in air and part of them transferred to the corresponding pyrrolo[3,2-*b*]-quinolones **12a–e**. Therefore, the conversation of **11a–e** to **12a–e** in near quantitative yields was accomplished through refluxing in ethanol with K_2_CO_3_ (Scheme 4, Table 3). 

**Table 3:** Synthesis of **11a–e** and **12a–e**.

Entry	10	R^2^	Product/yield (%)	Product/yield (%)
1	**10a**	H	**11a** (96)	**12a** (98)
2	**10b**	8-CH_3_	**11b** (94)	**12b** (97)
3	**10c**	8-OCH_3_	**11c** (94)	**12c** (96)
4	**10d**	8-Br	**11d** (95)	**12d** (96)
5	**10e**	6-Cl	**11e** (94)	**12e** (98)

## 3. Experimental

### 3.1. General

All melting points were measured on a Büchi apparatus and were not corrected. IR spectra (KBr pellets, 400–4,000 cm^−1^) were recorded on a Bruker VECTOR 22 FTIR spectrophotometer. ^1^H- and ^13^C-NMR spectra were recorded on a Brucker Avance DMX500 NMR spectrometer (500 and 125 MHz, respectively) using CDCl_3_, CD_3_OD, D_2_O, or DMSO-d_6_ as solvents with TMS as an internal standard. Elemental analyses were determined with a Thermo-Finnigan Flash EA 1112 elemental analyzer. ESI-HRMS spectra were measured with a Bruker Daltonics Apex β 7.0 FT-ICR MS instrument. Mass spectra (MS, ESI positive) were recorded on an Esquire-LC-00075 spectrometer.

### 3.2. Synthesis of Substituted tert-Butyl 3,4-dihydro-1H-pyrido[4,3-b]indole-2(5H)-carboxylates **8a–e**

A mixture of substituted phenylhydrazine hydrochloride (6.9 mmol) and 4,4-piperidinediol hydrochloride (8.5 mmol) in aqueous HCl solution (20 mL, 2.0 mol/L) was stirred at 60 °C for 16 h. After cooling to room temperature, the mixture was basified to pH > 12 with 25% NaOH solution. The precipitate formed was filtered off, washed with water and petroleum ether, and dried under reduced pressure to give the corresponding substituted 1,2,3,4-tetrahydro-γ-carbolines, which were used in the next step without further purification. To a solution of substituted 1,2,3,4-tetrahydro-γ-carboline in THF (20 mL), di-*tert*-butyl dicarbonate (8.2 mmol) was added at 0 °C, and the mixture was stirred at rt for 2 h. The solvent was removed under reduced pressure to give the crude product. Purification was performed by column chromatography on silica gel using ethyl acetate/petroleum ether (boiling range 60–90 °C) (1:2, v/v) as eluent to afford compounds **8a–e**.

*tert-Butyl 3,4-dihydro-1H-pyrido[4,3-b]indole-2(5H)-carboxylate *(**8a**) [16]. White solid (1.65 g, 88% yield in two steps); mp 145–146 °C; IR: ν 3,306, 3,065, 2,977, 2,916, 2,838, 1,655, 1,465, 1,428, 1,361, 1,161, 749, 663 cm^−1^; ^1^H-NMR (CDCl_3_): δ 7.97 (s, 1H), 7.46 (d, *J *= 7.5 Hz, 1H), 7.31 (d, *J *= 8.0 Hz, 1H), 7.15 (t, *J *= 7.5 Hz, 1H), 7.10 (t, *J *= 7.5 Hz, 1H), 4.64 (s, 2H), 3.82 (m, 2H), 2.82 (t, *J *= 5.5 Hz, 2H), 1.51 (s, 9H); HRMS calculated for C_16_H_21_N_2_O_2_ [M+H]^+^: 273.1598, found: 273.1596.

*tert-Butyl 8-methyl-3,4-dihydro-1H-pyrido[4,3-b]indole-2(5H)-carboxylate *(**8b**) [17]. White solid (1.68 g, 85% yield in two steps); mp 167–168 °C; IR: ν 3,307, 3,012, 2,974, 2,918, 2,839, 1,665, 1,475, 1,431, 1,360, 1,170, 761, 674 cm^−1^; ^1^H-NMR (CDCl_3_): δ 7.81 (s, 1H), 7.23 (s, 1H), 7.19 (d, *J *= 8.0 Hz, 1H), 6.97 (d, *J *= 8.0 Hz, 1H), 4.61 (s, 2H), 3.81 (br s,2H), 2.80 (br s, 2H), 2.44 (s, 3H), 1.50 (s, 9H); HRMS calculated for C_17_H_23_N_2_O_2_ [M+H]^+^: 287.1754, found: 287.1753.

*tert-Butyl 8-methoxy-3,4-dihydro-1H-pyrido[4,3-b]indole-2(5H)-carboxylate *(**8c**) [18]. White solid (1.81g, 84% yield in two steps); mp 170–171 °C; IR: ν 3,267, 2,972, 2,930, 2,840, 1,657, 1,472, 1,428, 1,368, 1,143, 865 cm^−1^; ^1^H-NMR (CDCl_3_): δ 7.77 (s, 1H), 7.20 (d, *J *= 8.5 Hz, 1H), 6.89 (d, *J *= 2.5 Hz, 1H), 6.80 (dd, *J_1_* = 8.5 Hz, *J_2_* = 2.5 Hz, 1H), 4.61 (s, 2H), 3.86 (s, 3H), 3.81 (t, *J *= 5.5 Hz, 1H), 2.81 (t, *J *= 5.5 Hz, 1H), 1.51 (s, 9H); HRMS calculated for C_17_H_23_N_2_O_3_ [M+H]^+^: 303.1703, found: 303.1702. 

*tert-Butyl 8-bromo-3,4-dihydro-1H-pyrido[4,3-b]indole-2(5H)-carboxylate *(**8d**) [19]. Slightly yellow solid (1.99 g, 82% yield in two steps); mp 177–178 °C (lit 177–179 °C); IR: ν 3,287, 3,007, 2,973, 2,926, 2,871, 2,837, 1,668, 1,589, 1,476, 1,461, 1,427, 1,361, 1,291, 1,232, 1,164, 675 cm^−1^; ^1^H-NMR (CDCl_3_): δ 8.11 (s, 1H), 7.55 (s, 1H), 7.21 (d, 1H, *J *= 8.5 Hz), 7.16 (d, *J *= 8.5 Hz, 1H), 4.58 (s, 2H), 3.79 (t, *J *= 5.0 Hz, 1H), 2.81 (t, *J *= 5.0 Hz, 1H), 1.51 (s, 9H); HRMS calculated for C_16_H_20_BrN_2_O_2_ [M+H]^+^: 351.0703, found: 351.0701.

*tert-Butyl 6-chloro-3,4-dihydro-1H-pyrido[4,3-b]indole-2(5H)-carboxylate *(**8e**)*.* White solid (1.84 g, 87% yield in two steps); mp 185–186 °C; IR: ν 3,262, 2,973, 2,924, 2,850, 1,671, 1,625, 1,469, 1,428, 1,361, 1,253, 1,159, 771, 691 cm^−1^; ^1^H-NMR (CDCl_3_): δ 8.17 (s, 1H), 7.34 (d, *J *= 7.0 Hz, 1H), 7.14 (d, *J *= 7.5 Hz, 1H), 7.02 (dd, *J_1_* = 8.0 Hz, *J_2_* = 7.5 Hz, 1H), 4.62 (s, 2H), 3.82 (br s, 2H), 2.85 (br s, 2H), 1.51 (s, 9H); ^13^C NMR (125 MHz, CDCl_3_): δ 155.1, 133.0, 126.9, 120.8, 120.2, 116.1, 108.4, 80.0, 41.2, 40.4, 28.4, 23.4; HRMS calculated for C_16_H_20_ClN_2_O_2_ [M+H]^+^: 307.1208, found: 307.1205.

### 3.3. Synthesis of Other 2-Substituted-1,2,3,4-tetrahydro-γ-carbolines **8f–i**

Acetyl chloride (2.4 mmol) was added dropwise at 0 °C to a solution of 1,2,3,4-tetrahydro-γ-carboline (2.0 mmol) in anhydrous CH_2_Cl_2_ (20 mL) containing triethylamine (1.0 mL). The reaction mixture stirred at room temperature until the 1,2,3,4-tetrahydro-γ-carboline disappeared (as monitored by TLC). The solvent and excess reagents were removed under reduced pressure to give the crude product. Purification was performed by column chromatography on silica gel using ethyl acetate/petroleum ether (boiling range 60–90 °C) (2:1, v/v) as eluent to afford **8f**. The acetyl chloride was replaced by benzoyl chloride, benzyl chloroformate or *p*-toluenesulfonyl chloride for the synthesis of **8g**, **8h** and **8i**, respectively.

*1-(3,4-Dihydro-1H-pyrido[4,3-b]indol-2(5H)-yl)ethanone *(**8f**) [20]. Off-white solid (360 mg, 84% yield); mp 256–257 °C (lit. 256–257 °C); IR: ν 3,143, 3,060, 2,945, 2,866, 1,604, 1,447, 1,359, 1,228, 1,146, 746 cm^−1^; ^1^H-NMR (DMSO-d_6_) analysis revealed the presence of two rotamers present in a 1.5:1 ratio: δ 10.89 (s, 1H, major rotamer), 10.87 (s, 1H, minor rotamer), 7.40 (m, 1H), 7.28 (dd, *J_1_* = 8.0 Hz, *J_2_* = 3.0 Hz, 1H), 7.01–7.05 (m, 1H), 6.95 (dd, *J_1_* = 14.0, *J_2_* = 7.0 Hz, 1H), 4.64 (s, 2H, minor rotamer), 4.62 (s, 2H, major rotamer), 3.83 (t, *J *= 6.0 Hz, 2H, minor rotamer), 3.76 (t, *J *= 6.0 Hz, 2H, major rotamer), 2.86 (t, *J *= 5.5 Hz, 2H, major rotamer), 2.74 (t, *J *= 5.5 Hz, 2H, minor rotamer), 2.13 (s, 3H, major rotamer), 2.12 (s, 3H, minor rotamer); ESI-MS: *m/z* 215.07 [M+H]^+^.

*(3,4-Dihydro-1H-pyrido[4,3-b]indol-2(5H)-yl)(phenyl)methanone *(**8g**). Off-white solid (492 mg, 89% yield); mp 204–206 °C; IR: ν 3,198, 3,059, 2,903, 2,840, 1,612, 1,577, 1,444, 1,237, 743, 709 cm^−1^; ^1^H NMR (CDCl_3_) analysis revealed the presence of two rotamers present in a 1.1:1 ratio: δ 8.05 (br s, 1H, major rotamer), 8.02 (br s, 1H, minor rotamer), 7.45–7.53 (m, 5H), 7.25–7.31 (m, 2H), 7.03–7.18 (m, 2H), 4.97 (s, 2H, minor rotamer), 4.66 (s, 2H, major rotamer), 4.16 (br s, 2H, major rotamer), 3.75 (br s, 2H, minor rotamer), 2.98 (br s, 2H, major rotamer), 2.86 (br s, 2H, minor rotamer); ^13^C-NMR (125 MHz, CDCl_3_): δ 171.4 (major rotamer), 171.1 (minor rotamer), 136.0 (major rotamer), 135.8 (minor rotamer), 132.4, 130.8, 129.9 (major rotamer), 129.7 (minor rotamer), 128.6 (major rotamer), 128.5 (minor rotamer), 127.1 (major rotamer), 126.7 (minor rotamer), 125.5 (minor rotamer), 124.9 (major rotamer), 121.7 (minor rotamer), 121.6 (major rotamer), 119.6 (minor rotamer), 110.8 (major rotamer), 106.9 (minor rotamer), 106.6 (major rotamer), 45.5 (major rotamer), 45.1 (minor rotamer), 40.3 (major rotamer), 40.2 (minor rotamer), 24.2 (minor rotamer), 23.1 (major rotamer); HRMS (ESI) calculated for C_18_H_17_N_2_O [M+H]^+^: 277.1335, found: 277.1339.

*Benzyl 3,4-dihydro-1H-pyrido[4,3-b]indole-2(5H)-carboxylate *(**8h**) [21]. White solid (551 mg, 90% yield); mp 115–117 °C; IR: ν 3,387, 3,019, 2,931, 2,858, 1,701, 1,465, 1,434, 1,240, 1,151, 752 cm^−1^; ^1^H-NMR (CDCl_3_): δ 7.91 (br s, 1H), 7.31–7.43 (m, 7H), 7.15 (t, *J *= 7.5 Hz, 1H), 7.08 (t, *J *= 7.5 Hz, 1H), 5.20 (s, 2H), 4.73 (s, 2H), 3.87–3.90 (m, 2H), 2.82–2.85 (m, 2H); HRMS (ESI) calculated for C_19_H_19_N_2_O_2_ [M+H]^+^: 307.1441, found: 307.1440.

*2-Tosyl-2,3,4,5-tetrahydro-1H-pyrido[4,3-b]indole *(**8i**) [13]. Light yellow solid (574 mg, 88% yield); mp 187–188 °C (lit. 187–189 °C) ^1^H-NMR (CDCl_3_): 7.86 (br s, 1H), 7.50 (d, *J* = 8.0 Hz, 2H), 7.37 (d, *J *= 7.5 Hz, 1H), 7.31 (d, *J *= 8.0 Hz, 2H), 7.26 (d, *J *= 5.5 Hz, 1H), 7.14 (t, *J *= 7.5 Hz, 1H), 7.08 (t, *J *= 7.5 Hz, 1H), 4.38 (s, 2H), 3.51 (t, *J *= 6.0 Hz, 1H), 2.87 (t, *J *= 6.0 Hz, 1H), 2.41 (s, 3H).

### 3.4. Synthesis of Substituted Dihydropyrrolo[3.2-b]quinolones **10a–i**

Substituted-1,2,3,4-tetrahydro-γ-carbolines (**8a–i**, 0.5 mmol), sodium hydroxide powder (1.0 mmol) and anhydrous DMF (5.0 mL) were placed in a 25 mL round-bottomed flask equipped with a calcium chloride drying tube. The mixture was stirred for 5–8 h at room temperature until the substrate disappeared. The reaction mixture was concentrated *in vacuo* and the residue was partitioned between water (10 mL) and ethyl acetate (20 mL). The organic layer was separated and the aqueous layer was further extracted with ethyl acetate (2 × 20 mL). The combined organic layer was washed with brine (20 mL), dried over Na_2_SO_4_ and evaporated *in vacuo*. The crude product was purified by column chromatography on silica gel using ethyl acetate/petroleum ether (boiling range 60–90 °C) (1:1, v/v) as eluent to give **10a-i**.

*tert-Butyl 9-oxo-2,3,4,9-tetrahydro-1H-pyrrolo[3,2-b]-quinoline-1-carboxylate *(**10a**). Off-white solid (135 mg, 94% yield); mp 159–161 °C; IR: ν 3,071, 2,984, 2,931, 2,626, 1,687, 1,621, 1,442, 1,380, 1,153, 1,031, 862, 753 cm^−1^; ^1^H-NMR (CDCl_3_): δ 12.47 (s, 1H), 8.23 (d, *J *= 7.5 Hz, 1H), 7.84 (d, *J *= 8.5 Hz, 1H), 7.56 (dt, *J_1_* = 8.5 Hz, *J_2_* = 1.8 Hz, 1H), 7.42 (t, *J *= 7.5 Hz, 1H), 4.02 (t, *J *= 8.5 Hz, 1H), 3.31 (t, *J *= 8.5 Hz, 1H), 1.59 (s, 9H); ^13^C-NMR (CDCl_3_): δ 157.5, 155.1, 147.1, 145.9, 128.4, 127.6, 124.9, 122.5, 121.5, 117.2, 83.7, 46.4, 30.5, 29.3; ESI-MS: *m/z* 287.02 [M+H]^+^; Anal. Calcd for C_16_H_18_N_2_O_3_: C, 67.12; H, 6.34; N, 9.78; found: C, 67.43; H, 6.48; N, 9.53.

*tert-Butyl 7-methyl-9-oxo-2,3,4,9-tetrahydro-1H-pyrrolo[3,2-b]quinoline-1-carboxylate *(**10b**). Off-white solid (138 mg, 92% yield); mp 185–186 °C; IR: ν 2,977, 2,934, 2,646, 1,665, 1,620, 1,569, 1,453, 1,387, 1,150, 1,034, 857 cm^−1^; ^1^H-NMR (CDCl_3_): δ 12.40 (s, 1H), 7.99 (s, 1H), 7.74 (d, *J *= 8.5 Hz, 1H), 7.38 (dd, *J_1_* = 8.5 Hz, *J_2_* = 2.0 Hz, 1H), 3.99 (t, *J *= 8.5 Hz, 1H), 3.27 (t, *J *= 8.5 Hz, 1H), 2.51 (s, 3H), 1.59 (s, 9H); ^13^C-NMR (CDCl_3_): δ 156.5, 155.1, 145.6, 145.5, 134.7, 130.6, 127.3, 121.4, 121.3, 171.2, 83.6, 46.4, 30.4, 28.3, 21.6; ESI-MS: *m/z* 300.94 [M+H]^+^; Anal. Calcd for C_17_H_20_N_2_O_3_: C, 67.98; H, 6.71; N, 9.33; found: C, 67.95; H, 6.82; N, 9.41.

*tert-Butyl 7-methoxy-9-oxo-2,3,4,9-tetrahydro-1H-pyrrolo[3,2-b]quinoline-1-carboxylate *(**10c**). Off-white solid (141 mg, 89% yield); mp 151–152 °C; IR: ν 3,103, 2,977, 2,931, 2,605, 1,685, 1,659, 1,617, 1,446, 1,392, 1,148, 1,030, 856 cm^−1^; ^1^H-NMR (CDCl_3_): δ 12.46 (s, 1H), 7.75 (d, *J *= 9.0 Hz, 1H), 7.50 (s, 1H), 7.21 (d, *J *= 9.0 Hz, 1H), 4.01 (t, *J *= 8.0 Hz, 1H), 3.93 (s, 3H), 3.28 (t, *J *= 8.0 Hz, 1H), 1.59 (s, 9H); ^13^C-NMR (CDCl_3_): δ 157.0, 155.2, 154.9, 145.0, 142.8, 129.0, 122.2, 120.6, 117.5, 100.6, 83.7, 55.5, 46.6, 30.2, 28.3; ESI-MS: *m/z* 316.88 [M+H]^+^; Anal. Calcd for C_17_H_20_N_2_O_4_: C, 64.54; H, 6.37; N, 8.86; found: C, 64.30; H, 6.48; N, 8.64.

*tert-Butyl 7-bromo-9-oxo-2,3,4,9-tetrahydro-1H-pyrrolo[3,2-b]quinoline-1-carboxylate *(**10d**). Light yellow solid (168 mg, 92% yield); mp 179–180 °C; IR: ν 2,979, 2,928, 2,655, 1,664, 1,615, 1,440, 1,379, 1,153, 1,032, 851 cm^−1^; ^1^H-NMR (CDCl_3_): δ 12.49 (s, 1H), 8.37 (d, *J *= 2.0 Hz, 1H), 7.69 (d, *J *= 9.0 Hz, 1H), 7.61 (dd, *J_1_* = 9.0 Hz, *J_2_* = 2.0 Hz, 1H), 4.04 (t, *J *= 8.5 Hz, 2H), 3.30 (t, *J *= 8.5 Hz, 2H), 1.59 (s, 9H); ^13^C-NMR (CDCl_3_): δ 158.0, 155.1, 145.6, 144.9, 131.7, 129.3, 124.9, 122.9, 118.8, 117.8, 84.0, 46.4, 30.4, 28.3; ESI-MS: *m/z* 364.86 [M+H]^+^. Anal. Calcd for C_16_H_17_BrN_2_O_3_: C, 52.62; H, 4.69; N, 7.67; found: C, 52.90; H, 4.72; N, 7.50.

*tert-Butyl 5-chloro-9-oxo-2,3,4,9-tetrahydro-1H-pyrrolo[3,2-b]quinoline-1-carboxylate *(**10e**). White solid (149 mg, 93% yield); mp 163–164 °C; IR: ν 2,974, 2,924, 2,672, 1,654, 1,621, 1,441, 1,371, 1,311, 1,157, 1,036, 827, 754 cm^−1^; ^1^H-NMR (CDCl_3_): δ 12.61 (s, 1H), 8.15 (d, *J *= 8.5 Hz, 1H), 7.66 (d, *J *= 7.5 Hz, 1H), 7.31 (t, *J_1_* = 8.0 Hz, *J_2_* = 8.0 Hz, 1H), 4.04 (t, *J *= 8.5 Hz, 2H), 3.40 (t, *J *= 8.5 Hz, 2H), 1.59 (s, 9H); ^13^C-NMR (CDCl_3_): δ 158.5, 155.2, 146.1, 143.3, 131.6, 128.6, 124.7, 123.1, 121.7, 117.9, 84.0, 46.5, 30.8,28.3; ESI-MS: *m/z* 320.87 [M+H]^+^; Anal. Calcd for C_16_H_17_ClN_2_O_3_: C, 59.91; H, 5.34; N, 8.73; found: C, 60.31; H, 5.50; N, 8.82.

*1-Acetyl-2,3-dihydro-1H-pyrrolo[3,2-b]quinolin-9(4H)-one *(**10f**). Light yellow solid (99 mg, 87% yield); mp 196–197 °C; IR: ν 3,057, 2,935, 2,363, 1,606, 1,455, 923, 774 cm^−1^; ^1^H-NMR (CDCl_3_): δ 13.14 (s, 1H), 8.26 (d, *J *= 8.5 Hz, 1H), 7.85 (d, *J *= 8.5 Hz, 1H), 7.61 (t, *J *= 7.0 Hz, 1H), 7.45 (t, *J *= 7.5 Hz, 1H), 4.10 (t, *J *= 8.5 Hz, 2H), 3.39 (t, *J *= 8.5 Hz, 2H), 2.33 (s, 3H); ^13^C-NMR (CDCl_3_): δ 170.3, 157.2, 148.2, 147.8, 129.3, 127.6, 125.1, 123.3, 121.4, 117.8, 48.0, 30.7, 23.0; ESI-MS: *m/z* 229.13 [M+H]^+^; Anal. Calcd for C_13_H_12_N_2_O_2_: C, 68.41; H, 5.30; N, 12.27; found: C, 68.67; H, 5.46; N, 11.94.

*1-Benzoyl-2,3-dihydro-1H-pyrrolo[3,2-b]quinolin-9(4H)-one *(**10g**). Light yellow solid (129 mg, 89% yield); mp 213–214 °C; IR: ν 3,075, 2,995, 2,951, 1,639, 1,582, 1,508, 1,462, 1,389, 1,323, 766 cm^−1^; ^1^H-NMR (CDCl_3_): δ 12.12 (s, 1H), 8.33 (d, *J *= 8.0 Hz, 1H), 7.90 (d, *J *= 8.0 Hz, 1H), 7.64–7.66 (m, 3H), 7.48–7.58 (m, 4H), 4.14 (t, *J *= 8.0 Hz, 2H), 3.32 (t, *J *= 8.0 Hz, 2H); ^13^C-NMR (CDCl_3_): δ 170.3, 157.6, 149.0, 147.8, 134.6, 131.3, 129.6, 128.8, 127.5, 127.2, 125.4, 123.3, 121.7, 118.4, 60.0, 31.3; ESI-MS: *m/z* 291.14 [M+H]^+^; Anal. Calcd for C_18_H_14_N_2_O_2_: C, 74.47; H, 4.86; N, 9.65; found: C, 74.21; H, 4.90; N, 9.58.

*Benzyl-9-oxo-2,3,4,9-tetrahydro-1H-pyrrolo[3,2-b]quinoline-1-carboxylate *(**10h**). Light yellow solid (138 mg, 86% yield); mp 153–154 °C; IR: ν 3,115, 2,963, 2,909, 1,669, 1,570, 1,501, 1,473, 1,161, 846, 775, 741 cm^−1^; ^1^H-NMR (CDCl_3_): δ 12.21 (s, 1H), 8.23 (d, *J *= 8.5 Hz, 1H), 7.84 (d, *J *= 8.5 Hz, 1H), 7.56–7.59 (m, 1H), 7.35–7.45 (m, 6H), 5.31 (s, 2H), 4.08 (t, *J *= 8.5 Hz, 2H), 3.31 (t, *J *= 8.5 Hz, 2H); ^13^C-NMR (CDCl_3_): δ 157.2, 155.4, 147.2, 146.0, 135.1, 128.7, 128.6, 128.4, 127.6, 125.1, 122.5, 121.4, 116.6, 69.1, 46.2, 30.4; ESI-MS: *m/z* 321.12 [M+H]^+^; Anal. Calcd for C_19_H_16_N_2_O_3_: C, 71.24; H, 5.03; N, 8.74; found: C, 71.10; H, 5.05; N, 8.59.

*1-Tosyl-2,3-dihydro-1H-pyrrolo[3,2-b]quinolin-9(4H)-one *(**10i**) [13]. Light yellow solid (65 mg, 38% yield); mp 207–208 °C (lit. 207–209 °C); ^1^H-NMR (DMSO-d_6_): 12.06 (s, 1H), 8.15 (d, *J *= 7.0 Hz, 1H), 7.65 (d, *J *= 8.0 Hz, 2H), 7.60 (m, 1H,), 7.46 (d, *J *= 9.0 Hz, 1H), 7.32–7.35 (m, 3H), 3.99 (t, *J *= 7.5 Hz, 2H), 2.48–2.50 (m, 2H), 2.36 (s, 3H).

### 3.5. Synthesis of Dihydropyrrolo[3,2-b]quinolone Hydrochlorides **11a–e**

2-Boc-dihydropyrrolo[3,2-b]quinolones (**10a–e**, 0.5 mmol) was dissolved in a mixture of HCl saturated ethyl acetate (5 mL) and methanol (5 mL) under nitrogen. The reaction mixture was refluxed for 6–8 h and cooled to room temperature, the precipitate was filtered and washed with cool ethyl acetate to afford **11a–e**.

*2,3-Dihydro-1H-pyrrolo[3,2-b]quinolin-9(4H)-one hydrochloride *(**11a**). Yellow solid (107 mg, 96% yield); mp > 250 °C; IR (KBr): ν 3,091, 2,983, 2,670, 1,635, 1,595, 1,536, 1,465, 1,418, 1,355, 1,266, 756, 682 cm^−1^. ^1^H NMR (500 MHz, D_2_O): δ 8.17 (d, 1H, *J *= 8.0 Hz), 7.80 (t, 1H, *J *= 8.0 Hz), 7.54–7.61 (m, 2H), 4.06 (t, *J *= 7.5 Hz, 2H), 3.58 (t, *J *= 7.5 Hz, 2H); ^13^C NMR (125 MHz, D_2_O): δ 170.2, 150.6, 139.6, 133.2, 125.1, 124.5, 124.0, 118.8, 114.8, 43.8, 28.5; HRMS (ESI) calculated for C_11_H_11_N_2_O [M+H]^+^: 187.0866, found: 187.0857.

*7-Methyl-2,3-dihydro-1H-pyrrolo[3,2-b]quinolin-9(4H)-one hydrochloride *(**11b**). Yellow solid (111 mg, 94% yield); mp > 250 °C; IR (KBr): ν 3,088, 2,988, 2,447, 1,633, 1,570, 1,528, 1,483, 1,418, 1,360, 829 cm^−1^; ^1^H NMR (500 MHz, D_2_O): δ 7.76 (s, 1H), 7.49 (dd, *J_1_* = 8.5 Hz, *J_2_* = 1.5 Hz, 1H), 7.33 (d, *J *= 8.5 Hz, 1H), 3.94 (t, *J *= 8.0 Hz, 2H), 3.44 (t, *J *= 8.0 Hz, 2H), 2.38 (s, 3H); ^13^C NMR (125 MHz, D_2_O): δ 169.6, 149.7, 137.4, 135.5, 134.5, 124.2, 122.6, 118.3, 114.6, 43.7, 28.3, 20.2; HRMS (ESI) calculated for C_12_H_12_N_2_NaO [M+Na]^+^: 223.0842, found: 223.0838.

*7-Methoxy-2,3-dihydro-1H-pyrrolo[3,2-b]quinolin-9(4H)-one hydrochloride *(**11c**). Yellow solid (119 mg, 94% yield); mp > 250 °C; IR (KBr): ν 2,813, 2,649, 2,460, 1,613, 1,509, 1,475, 1,348, 1,246, 853 cm^−1^; ^1^H NMR (500 MHz, D_2_O): δ 7.15 (d, *J *= 9.0 Hz, 1H), 7.11 (s, 1H), 7.03 (d, *J *= 9.0 Hz, 1H), 3.92 (t, *J *= 8.0 Hz, 2H), 3.72 (s, 3H), 3.40 (t, *J *= 8.0 Hz, 2H); ^13^C NMR (125 MHz, D_2_O): δ 168.9, 156.3, 149.1, 134.4, 125.6, 123.0, 120.2, 114.4, 103.4, 55.6, 43.9, 28.3; HRMS (ESI) calculated for C_12_H_13_N_2_O_2_ [M+H]^+^: 217.0971, found: 217.0964.

*7-Bromo-2,3-dihydro-1H-pyrrolo[3,2-b]quinolin-9(4H)-one hydrochloride *(**11d**)*.* Brown solid (143 mg, 95% yield); mp > 250 °C; IR (KBr): ν 2,853, 2,757, 2,469, 1,629, 1,574, 1,518, 1,461, 852 cm^−1^; ^1^H NMR (500 MHz, D_2_O): δ 7.88 (s, 1H), 7.56 (d, *J *= 8.5 Hz, 1H), 7.20 (d, *J *= 9.0 Hz,1H), 3.98 (t, *J *= 8.0 Hz, 2H), 3.53 (t, *J *= 8.0 Hz, 2H); ^13^C NMR (125 MHz, D_2_O): δ 168.8, 151.0, 138.3, 135.5, 126.3, 125.8, 120.6, 117.9, 115.4, 43.7, 28.7; HRMS (ESI) calculated for C_11_H_10_BrN_2_O [M+H]^+^: 264.9971, found: 264.9969.

*5-Chloro-2,3-dihydro-1H-pyrrolo[3,2-b]quinolin-9(4H)-one hydrochloride *(**11e**). Slightly yellow solid (121 mg, 94% yield); mp > 250 °C; IR (KBr): ν 3,084, 2,915, 2,483, 1,630, 1,594, 1,422, 750 cm^−1^; ^1^H NMR (500 MHz, D_2_O): δ 7.80 (d, *J *= 8.5 Hz, 1H), 7.62 (d, *J *= 7.5 Hz, 1H), 7.22 (t, 1H, *J *= 8.0 Hz), 3.95 (t, *J *= 8.0 Hz, 2H), 3.47 (t, *J *= 8.0 Hz, 2H); ^13^C NMR (125 MHz, D_2_O): δ 170.5, 151.8, 136.9, 133.7, 126.8, 125.8, 124.0, 123.4, 116.6, 49.5, 44.4, 29.7; HRMS (ESI) calculated for C_11_H_10_ClN_2_O [M+H]^+^: 221.0476, found: 221.0472.

### 3.6. Synthesis of Pyrrolo[3.2-b]quinolones (**12a–e**)

Dihydropyrrolo[3,2-b]quinolone hydrochloride (**11a–e**, 0.5 mmol) was mixed with potassium carbonate (1.0 mmol) in ethanol (10 mL) and refluxed for 4–6 h. The mixture was concentrated *in vacuo*, the residue was purified by column chromatography on silica gel using ethyl acetate/petroleum ether (boiling range 60–90 °C) (4:1, v/v) as eluent to give **12a–e**.

*1H-Pyrrolo[3,2-b]quinolin-9(4H)-one *(**12a**) [12]. Light yellow solid (90 mg, 98% yield); mp > 250 °C; IR: ν 3,164, 3,030, 2,900, 1,690, 1,637, 1,595, 1,518, 1,459, 1,413, 750, 669 cm^−1^; ^1^H-NMR (DMSO-d_6_): δ 8.25 (d, *J *= 7.0 Hz, 1H), 7.56 (dt, *J_1_* = 8.5 Hz, *J_2_* = 1.5 Hz, 1H), 7.49 (d, *J *= 8.0 Hz, 1H), 7.37 (t, *J *= 3.0 Hz, 1H), 7.15 (t, *J *= 7.5 Hz, 1H), 6.20 (t, *J *= 2.5 Hz, 1H); HRMS (ESI) calculated for C_22_H_17_N_4_O_2_ [2M+H]^+^: 369.1346, found: 369.1345.

*7-Methyl-1H-pyrrolo[3,2-b]quinolin-9(4H)-one *(**12b**) [12]. Yellow solid (96 mg, 97% yield); mp > 250 °C; IR: ν 3,170, 3,029, 2,930, 1,584, 1,520, 1,475, 1,404, 1,356, 1,302, 1,144, 787 cm^−1^; ^1^H-NMR (DMSO-d_6_): δ 11.77 (s, 1H), 11.66 (s, 1H), 8.05 (s, 1H), 7.36–7.42 (m, 3H), 6.18 (s, 1H), 2.41 (s, 3H); HRMS (ESI) calculated for C_12_H_11_N_2_O [M+H]^+^: 199.0866, found: 199.0864.

*7-Methoxy-1H-pyrrolo[3,2-b]quinolin-9(4H)-one *(**12c**) [12]. Yellow solid (103 mg, 96% yield); mp 250 °C; IR: ν 3,173, 3,015, 2,930, 1,583, 1,522, 1,478, 1,403, 1,356, 1,270, 756 cm^−1^; ^1^H-NMR (CD_3_OD): δ 7.76 (d, *J *= 2.5 Hz, 1H), 7.45–7.47 (m, 2H), 7.23 (dd, *J_1_* = 9.0 Hz, *J_2_* = 2.5 Hz, 1H), 6.26 (d, *J *= 3.0 Hz, 1H), 3.86(s, 3H); ^13^C-NMR (CD_3_OD): δ 167.3, 155.9, 138.7, 136.0, 131.1, 123.7, 123.2, 121.7, 120.1, 105.0, 95.6, 56.1; HRMS (ESI) calculated for C_12_H_11_ClN_2_O_2_ [M+H]^+^: 215.0815, found: 215.0814.

*7-Bromo-1H-pyrrolo[3,2-b]quinolin-9(4H)-one *(**12d**) [12]. Yellow solid (126 mg, 96% yield); mp > 250 °C; IR: ν 3,150, 2,924, 1,636, 1,594, 1,514, 1,454, 1,262, 1,153, 1,031, 810, 755 cm^−1^; ^1^H-NMR (CD_3_OD): δ 8.47 (d, *J *= 2.0 Hz, 1H), 7.65 (dd, 1H, *J_1_* = 9.0 Hz, *J_2_* = 2.0 Hz), 7.48 (d, 1H, *J *= 3.0 Hz), 7.44 (d, 1H, *J *= 8.5 Hz), 6.29 (d, 1H, *J *= 3.0 Hz); ^13^C-NMR (CD_3_OD): δ 166.9, 139.5, 138.8, 134.8, 131.3, 128.7, 124.1, 121.7, 120.4, 95.9; HRMS (ESI) calculated for C_11_H_7_BrN_2_O [M+H]^+^: 262.9815, found: 262.9814.

*5-Chloro-1H-pyrrolo[3,2-b]quinolin-9(4H)-one *(**12e**)*. *Yellow solid (107 mg, 98% yield); mp > 250 °C; IR: ν 3,149, 3,023, 2,916, 1,627, 1,583, 1,514, 1,442, 1,355, 1,103, 774 cm^−1^; ^1^H-NMR (CD_3_OD): δ 8.29 (d, *J *= 8.5 Hz, 1H), 7.63 (d, *J *= 7.5 Hz, 1H), 7.44 (d, *J *= 3.0 Hz, 1H), 7.13 (dd, *J_1_* = 8.0 Hz, *J_2_* = 7.5 Hz, 1H), 6.40 (d, *J *= 3.0 Hz, 1H); ^13^C-NMR (CD_3_OD): δ 168.2, 139.1, 137.7, 132.4, 131.6, 126.2, 124.9, 122.9, 122.1, 121.9, 97.3; HRMS (ESI) calculated for C_11_H_7_ClN_2_NaO [M+Na]^+^; 241.0139, found: 241.0135.

## 4. Conclusions

In summary, we have reported a convenient and efficient synthesis of dihydropyrrolo[3,2-b]-quinolones and pyrrolo[3,2-b]quinolones via Winterfeldt oxidation of 1,2,3,4-tetrahydro-γ-carbolines. The results suggest that electron-withdrawing groups on the 2-position of 1,2,3,4-tetrahydro-γ-carbolines are necessary for this transformation. Apart from experimental simplicity and excellent yields, it is noteworthy that this is the first report on the classical Winterfeldt oxidation of substituted 1,2,3,4-tetrahydro-γ-carbolines.
